# Hemofiltration induces generation of leukocyte-derived CD31+/CD41− microvesicles in sepsis

**DOI:** 10.1186/s13613-017-0312-3

**Published:** 2017-09-04

**Authors:** Georg Franz Lehner, Ulrich Harler, Clemens Feistritzer, Viktoria Maria Haller, Julia Hasslacher, Romuald Bellmann, Michael Joannidis

**Affiliations:** 1 Division of Intensive Care and Emergency Medicine, Department of Internal Medicine, Medical University Innsbruck, Anichstrasse 35, 6020 Innsbruck, Austria; 2 Department of Internal Medicine V - Haematology and Oncology, Medical University Innsbruck, Anichstrasse 35, 6020 Innsbruck, Austria

**Keywords:** Sepsis, Microvesicles, Microparticles, Hemofiltration, Continuous veno-venous hemofiltration, Renal replacement therapy, Tissue factor, Leukocytes, Platelets

## Abstract

**Background:**

Microvesicles (MV) are extracellular vesicles known to be associated with cellular activation and inflammation. Hemofiltration is an effective blood purification technique for patients with renal failure and possibly also eliminates inflammatory mediators in the setting of sepsis. On the other hand, proinflammatory stimuli are induced by blood contacting the artificial membrane during extracorporeal blood purification. In chronic dialysis patients a systemic increase in MV has been described. The aim of the study was to investigate whether hemofilter passage of blood in continuous veno-venous hemofiltration (CVVH) alters MV composition and levels in critically ill patients with sepsis.

**Methods:**

Pre- and postfilter bloods as well as ultrafiltrate samples from intensive care unit patients with severe sepsis were obtained during CVVH with regional citrate anticoagulation. MV subtypes in blood were analyzed by high-sensitivity flow cytometry. Additionally, tissue factor (TF) levels and MV-associated TF activities as well as MV activities were quantified. All parameters were corrected for hemoconcentration applied during CVVH.

**Results:**

Twelve patients were analyzed. A significant increase in presumably mostly leukocyte-derived CD31+/CD41− MV (1.32 (1.09–1.93)-fold [median (25th–75th quartiles)], *p* = 0.021) was observed post- to prefilter, whereas platelet-derived MV as well as AnnexinV-binding MV were unaltered. Increments of AnnexinV+, CD42b+ and CD31+/CD41− MV post- to prefilter correlated with filtration fraction (FF) (all *p* < 0.05). Significant reductions in MV activity [0.72 (0.62–0.84)-fold, *p* = 0.002] and TF level [0.95 (0.87–0.99)-fold, *p* = 0.0093] were detected postfilter compared to prefilter. No MV activity was measurable in ultrafiltrate samples.

**Conclusions:**

Despite clearing a fraction of small PS-exposing MV CVVH does not eliminate larger MV. Concurrently, CVVH induces the release of CD31+/CD4− MV that indicate leukocyte activation during hemofilter passage in septic patients. Increments of several MV subtypes within the hemofilter correlate with FF, which supports common recommendations to keep FF low. A fraction of TF is being cleared by CVVH via ultrafiltration.

**Electronic supplementary material:**

The online version of this article (doi:10.1186/s13613-017-0312-3) contains supplementary material, which is available to authorized users.

## Background

Continuous veno-venous hemofiltration (CVVH) is a frequently used renal replacement therapy (RRT) modality in critically ill patients [1]. Although it is an effective and life-saving treatment in the presence of renal failure, its beneficial effect as sole supportive sepsis therapy is still controversial [2, 3]. It has been shown that hemofiltration is able to influence levels of circulating mediators such as inflammatory cytokines by adsorption or filtration [4]. However, these potentially favorable effects might be offset by proinflammatory stimuli that are induced by blood contacting an artificial membrane during extracorporeal blood purification itself [5–7]. This effect may be enhanced when heparin is used for anticoagulation and attenuated during regional citrate anticoagulation [8]. Microvesicles (MV) are extracellular vesicles sized between approximately 100 nm and 1 µm that are released by several cell types upon stimulation or apoptosis [9, 10]. They mediate pleiotropic inflammatory signals during sepsis [11] and are associated with the occurrence of disseminated intravascular coagulation [12]. In patients with chronic renal failure increased systemic levels of platelet-derived as well as procoagulatory AnnexinV-binding MV were found in blood following RRT [13]. However, it is not known whether this increase reflects secondary systemic effects of RRT or a MV release within the hemofilter.

The aim of this study was to examine the influence of blood membrane contact during a single hemofilter passage in CVVH with citrate anticoagulation on MV levels and composition in patients with severe sepsis.

## Methods

### Patients

Patients from a tertiary intensive care unit (ICU) that required RRT and fulfilled at least two systemic inflammatory response syndrome (SIRS) criteria [14] in the presence of a suspected or proven sepsis focus were prospectively enrolled in the study from January 2012 to March 2013. Patients were excluded if they were younger than 18 years, moribund, pregnant or breast-feeding. The study was approved by the Ethics Committee from the Medical University Innsbruck (protocol UN 2705a 244/4.20). Subjects provided written informed consent either prior to enrollment or post hoc. Vital parameters were obtained, routine laboratory values measured, as well as acute physiology and chronic health evaluation (APACHE II) score [15], simplified acute physiology score (SAPS II) [16] and sequential organ failure assessment score (SOFA) [17] computed.

### Hemofiltration and sample collection

RRT was conducted in CVVH mode by using a ST150 (Gambro Hospital Austria GmbH, Wiener Neudorf, Austria) hemofilter in a Prismaflex^®^ machine (Gambro Hospital Austria GmbH) with postdilution. Phoxilium^®^ was used as substitution fluid. Anticoagulation was performed as regional citrate anticoagulation with Prismocitrate^®^ 18/0, aiming at a postfilter iCa^2+^ level of 0.35–0.45 mmol/l. Prefilter blood was obtained from a port located after citrate and prefilter substitution fluid influx, and postfilter blood from a port located directly after the hemofilter. Blood was drawn in 3-ml S-Monovette^®^ tubes (Sarstedt, AG & Co., Nümbrecht, Germany) with 3.2% citrate. Ultrafiltrate and blood samples were immediately centrifuged at 20 °C in a Hettich Rotanta 46 RC centrifuge at 1550 g for 15 min with acceleration and deceleration set at 8. The supernatant plasma from blood samples was then further centrifuged at 13000 *g* for 2 min in a Hettich Micro R22 centrifuge set at maximum acceleration and deceleration, and platelet-free plasma (PFP) obtained. Samples were flash-frozen in liquid nitrogen and stored at −80 °C. Upon analysis samples were thawed at 37 °C in a water bath for 2 min and then kept on wet ice.

### Quantification of microvesicles by high-sensitivity flow cytometry

PFP was labeled for 30 min at room temperature with AnnexinV–FITC (1.5 µg/ml [final concentration]) to label phosphatidylserine (PS) on MV, CD31-PE (PECAM; platelet endothelial cell adhesion molecule; 0.39 µg/ml) which is present on MV from endothelial cells, platelets as well as leukocytes and CD42b-APC (GPIbα; glycoprotein Ib alpha; 0.78 µg/ml; all from BD Pharmingen, San Jose, CA) as well as CD41-PC7 (GPIIb; glycoprotein IIb; 0.78 µg/ml; Beckman-Coulter, Miami, FL) to label platelet-derived MV. AnnexinV and antibodies were filtered with a 0.1-µm filter (Millipore, Darmstadt, Germany), and PBS and AnnexinV-binding buffer with a 0.2-µm syringe filter (Sarstedt, Nümbrecht, Germany). Following the addition of CytoCount™ beads (DakoCytomation, Glostrup, Denmark) and 500 µl of AnnexinV-binding buffer (BD Pharmigen) specimens were measured with a Gallios™ flow cytometer with the threshold set at forward scatter (FS) by using Gallios™ Cytometry List Mode Data Acquisition and Analysis Software 1.2 (both Beckman Coulter, Bra, CA). Isotype controls prepared analogously were run in parallel to assess background signals of samples that showed potential artifacts in flow cytometry. These controls were labeled with AnnexinV–FITC and matched irrelevant antibodies of the same isotype and diluted in calcium-free phosphate-buffered saline (PBS from PAA Laboratories, Pasching, Austria) instead of AnnexinV-binding buffer. Artifact-containing parameters were excluded in subsequent analyses (Additional file 1: Fig. S1). Flow cytometric data were analyzed with Kaluza^®^, version 1.2 (Beckman Coulter). The gating strategy defined MV in a size range between 0.3 and 1.0 µm polystyrene bead-equivalents (LB3 and 89904 from Sigma-Aldrich, Saint Louis, MO) that showed a positivity concerning the aforementioned markers. The number of MV per µl PFP was determined by referring to CytoCount™ beads.

### Microvesicle activity and tissue factor measurements

PFP and ultrafiltrate samples were analyzed with three solid-phase-capturing assays as recommended by the manufacturers. The quantity of PS, here referred to as MV activity, which is also one aspect of the procoagulatory potential of MV, was measured with the Zymuphen MP activity assay (Hyphen BioMed, Neuville sur Oise, France). The amount of tissue factor was assessed by using the Imubind^®^ tissue factor ELISA (Sekisu Diagnostics, Stamford, CT). The procoagulatory ability of TF in the presence of PS on MV, here referred to as TF activity, was analyzed with the Zymuphen MP-TF assay (Hyphen BioMed).

### Calculation of hemofiltration flow rates and statistical analyses

Absolute amounts of microvesicles entering and leaving the hemofilter were calculated by multiplying the measured MV counts (MV/µl) with the plasma flow or ultrafiltrate flow at the distinct sampling site per minute:

$$\begin{aligned} {\text{Amount}}_{{{\text{prefilter}}}} & = C_{{{\text{meas}}}} \times \left( {{\text{BF}}\times \left( {1 - \frac{{{\text{HCT}}}}{{100}}} \right) + \frac{{{\text{CF}}}}{{60}}} \right) \times 1000 \\ {\text{Amount}}_{{{\text{postfilter}}}} & = C_{{{\text{meas}}}} \times \left( {BF\times\left( {1 - \frac{{{\text{HCT}}}}{{100}}} \right) + \frac{{{\text{CF}}}}{{60}} - \frac{{{\text{UF}}}}{{60}}} \right)\times 1000 \\ {\text{Amount}}_{{{\text{UF}}}} & = C_{{{\text{meas}}}} \times \frac{{{\text{UF}}\times 1000}}{{60}} \\ {\text{FF}} & = \frac{{{\text{UF}}}}{{{\text{BF}} \times \left( {1 - \frac{{{\text{HCT}}}}{{100}}} \right) \times 60 + {\text{CF}}}} \\ \end{aligned}$$

C_meas_—concentration of MV measured at specific port (MV/µl); Amount_prefilter_—amount of MV entering the hemofilter (MV/min); Amount_postfilter_—amount of MV leaving the hemofilter via blood (MV/min); Amount_UF_—amount of MV leaving the hemofilter via ultrafiltrate (MV/min); BF—blood flow (ml/min); CF—flow of citrate-containing solution (ml/h); UF—ultrafiltrate flow (ml/h), as determined by the Prismaflex^®^ machine; HCT—hematocrit (%); and FF—filtration fraction.

Statistical analyses were performed with GraphPad Prism^®^ version 5 (GraphPad Software Inc., La Jolla, CA) and SPSS^®^ version 23 (IBM, Armonk, NY). Data were tested for normality by using the Shapiro–Wilk test. For comparison of amounts before and after the hemofilter the Wilcoxon signed-rank test was used. Correlations were analyzed in SPSS by using the Spearman rho test. Quantitative data are presented as median and 25th–75th quartiles, if not indicated otherwise. P values below 0.05 were considered statistically significant.

## Results

### Patients

Twelve eligible patients from a tertiary ICU with severe sepsis or septic shock requiring RRT due to renal failure were included in the study. Blood culture was positive in six patients. Primary sepsis focus was the lung in six patients, urinary tract in two patients and soft tissue in one patient. In three patients no distinct focus could be determined in addition to a positive blood culture. Additional patient characteristics are presented in Table 1.

**Table 1:** Patient characteristics

Age (years)	64 (11)
Gender (% male)	83
SOFA	14 (4)
SAPS II	67 (16)
APACHE II	29 (6)
Vasopressor requirement (%)	92
Leukocyte count (G/l)	15 (10)
Platelet count (G/l)	127 (145)
CRP (mg/dl)	19.3 (9.6)
PCT (ng/l)	39.9 (75.3)
ICU survival (%)	33
Hospital survival (%)	25

SOFA and laboratory parameters refer to the day of blood draw. Metric variables are presented as mean (standard deviation)

*G/l* Giga (10^9^) cells per liter, *SOFA* sequential organ failure assessment score, *APACHE II* acute physiology and chronic health evaluation, *SAPS II* simplified acute physiology score, *CRP* C-reactive protein, *PCT* procalcitonin

Table 2 provides an overview of the hemofiltration settings.

**Table 2:** Hemofiltration settings at the time of blood draw presented as mean (standard deviation)

Blood flow (ml/min)	126 (9)
Citrate flow (ml/h)	1240 (102)
Substitution fluid flow (ml/h)	921 (304)
Patient fluid withdrawal (ml/h)	83 (83)
Ultrafiltrate flow (ml/h)	2249 (298)
Filter runtime at the time of sampling (h)	7 (7.5)
Filter survival (h)	59.6 (17.5)

### Microvesicles and tissue factor in pre- and postfilter blood samples

To examine whether hemofiltration alters counts of several MV subtypes in blood the absolute amounts of MV subtypes entering and leaving the hemofilter per time were compared. Ratios of the amounts of distinct parameters in blood that enter and leave the hemofilter are shown in Table 3.

**Table 3:** Ratios of the amounts leaving the filter/entering the filter via blood presented as median (quartiles)

Parameter	Ratio amount postfilter/prefilter blood	*p*
AnnexinV+	0.94 (0.84–1.93)	1.0
CD41+	1.00 (0.93–1.80)	0.7334
CD42b+	1.02 (0.70–1.41)	0.8501
CD31+/CD41−	*1.32 (1.09–1.93)*	*0.0210*
CD31+/CD42b−	1.01 (0.91–1.91)	0.4697
MV activity	*0.72 (0.62*–*0.84)*	*0.0020*
TF activity	1.05 (0.85–1.36)	0.5186
TF level	*0.95 (0.87*–*0.99)*	*0.0093*

*P* values refer to the comparison of absolute amounts/minute leaving to the amounts/minute entering the hemofilter *via* blood

**Fig. 1:**
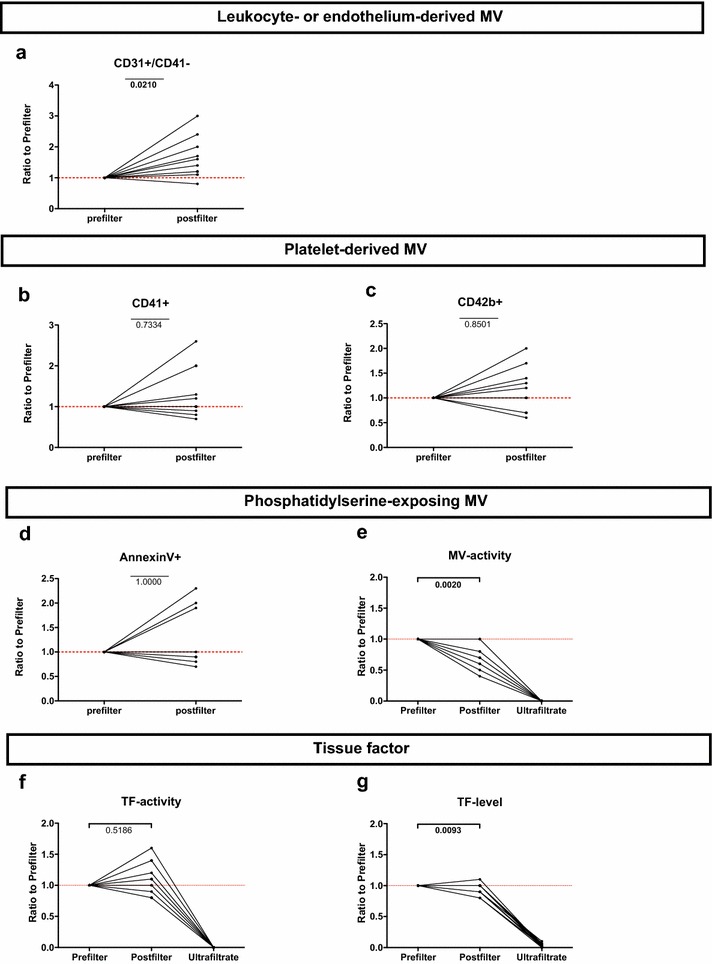
Postfilter-to-prefilter ratios of MV subsets, MV activity, TF activity and TF level. *P* values refer to the comparison of absolute amounts per minute pre- to postfilter

**Fig. 2:**
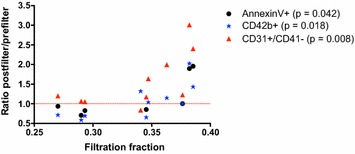
Correlations of MV subtypes (post- to prefilter Ratios) with filtration fraction

A significant increase in probably mostly leukocyte-derived CD31+/CD41− MV [8.45 × 10^6^ (3.37 × 10^6^ − 2.03 × 10^7^) vs. 1.19 × 10^7^ (5.49 × 10^6^ − 2.53 × 10^7^) MV/min; *p* = 0.021] (Fig. 1a) was detected after the hemofilter passage. However, no significant differences in counts of platelet-derived CD41+ and CD42b+ MV, AnnexinV+ MV or TF activity (all *p* > 0.05) were found (Fig. 1b–d, f). There were significant correlations between filtration fraction (FF) and the ratios of post- to prefilter MV (Fig. 2) of AnnexinV+, CD42b+ and CD31+/CD41− MV (Spearman’s correlation coefficient (r_s_) = 0.683, *p* = 0.042; r_s_ = 0.664, *p* = 0.018; r_s_ = 0.720, *p* = 0.008, respectively).

A measure of the total amount of PS-exposing MV, i.e., MV activity (Fig. 1e), revealed a statistically significant decrease during hemofilter [0.088 (0.027–0.243) vs. 0.067 (0.021–0.223) nmol/min; *p* = 0.002]. The comparison of TF levels (Fig. 1g) before and after the hemofilter showed a significant decrease after the hemofilter [27877 (22103–37793) vs. 25811 (18736–35164) pg/min, (*p* = 0.0376) pg/min; *p* = 0.0093)]. No significant correlation was detected between FF and alterations in MV activity, TF activity or TF level.

There were no correlations between platelet or leukocyte counts and the observed alterations of MV or TF during hemofilter passage. No associations between filter runtime and MV alterations were detected.

Three patients showing artifacts in AnnexinV+ MV counts and one patient with artifacts in measurements of MV activities were therefore excluded from analyses. However, this did not relevantly change statistics.

### Microvesicles and tissue factor in ultrafiltrate

To investigate whether MV or fragments of them are ultrafiltrated during hemofilter passage solid-phase-capturing assays were used to examine the ultrafiltrate, such as the MV activity assay to detect MV. This assay captures PS and is therefore able to detect also MV that are sized below the detection limit of high-sensitivity flow cytometry which is approximately 0.3 µm. However, no relevant MV activity could be detected in ultrafiltrate samples (i.e., absolute activities ≤0.05 nM). A remarkable fraction of TF (in average approximately 4.6% of the TF amount that enters the hemofilter) was found in the ultrafiltrate [1229 (408–2218) pg/min, corresponding to an average sieving coefficient of 0.12 (Fig. 1g). However, there was no relevant TF activity measurable in ultrafiltrate (i.e., absolute activities ≤0.05 pg/ml) (Fig. 1f).

## Discussion

This is the first study analyzing MV levels as well as TF in blood before and after the hemofilter in CVVH. The major findings of this study are that (1) CVVH clears small PS-exposing MV but not larger MV, (2) subsets of MV are generated within the hemofilter indicating leukocyte activation during hemofilter passage, (3) the extent of MV generation correlates with FF, and (4) a fraction of TF is being cleared via ultrafiltration.

### Effects of hemofiltration on microvesicle counts and composition

We could show that CVVH has differential effects on distinct MV subtypes. On the one hand, we observed a reduction in MV activity during hemofilter passage, but no change in AnnexinV+ MV. Although both parameters are measures of PS-exposing MV, they reflect different fractions of PS + MV: MV activity comprises the overall amount of PS + MV, whereas AnnexinV+ MV, measured with hs-FC, exclusively PS + MV that are larger than approximately 0.3 µm. Thus, our data indicate that CVVH clears a fraction small PS-exposing MV. Since no MV activity was measurable in the ultrafiltrate, we assume mechanisms other than filtration such as adsorption being responsible for the clearance of small MV. The findings from our clinical study are compatible with a study from Abdelhafeez et al. [18] who analyzed the effects of CVVH on MV counts in an ex vivo model of CVVH and reported a reduction in MV over a period of 70 min, as shown for endothelium-derived MV. In line with our findings, they propose that mechanisms other than filtration seem to be responsible for the reduction in MV [18]. In conclusion, our data suggest that CVVH clears a fraction of small PS-exposing MV from blood of septic patients.

On the other hand, we found a significantly higher amount of CD31+/CD41− MV after the hemofilter. According to their surface epitope pattern this MV subtype can either be derived from leukocytes or endothelial cells. However, CD31+/CD41− MV that are generated within the hemofilter are probably predominantly leukocyte derived. There are two aspects corroborating this assumption: Firstly, recent data suggest that the majority of MV exposing this not endothelium-specific CD31+/CD41− pattern are predominantly not derived from endothelial cells, but rather from leukocytes in patients with septic shock [19]. Secondly, it is evident that CD31+/CD41− MV that are released during a single hemofilter passage are originating from circulating cells. Despite low levels of circulating endothelial cells (CEC) present in patients with septic shock (approximately between 10 and 30 cells per ml) [20] it is likely that the observed significant increase in CD31+/CD41− MV is rather produced by leukocytes which are around half a million times more abundant than CEC. Our findings and conclusions are in accordance with a study from Kozek-Langenecker et al. [21] who directly assessed activation status of cells. Likewise, they report activation of leukocytes and also platelets within the hemofilter during CVVH with heparin anticoagulation [21]. In line with this, Faure et al. [13] found even higher systemic counts of leukocyte- and platelet-derived MV in patients with chronic renal failure that were dialyzed with heparin anticoagulation compared to patients without dialysis. Moreover, they observed a pronounced systemic increase in CD41+ platelet-derived MV and also in AnnexinV+ MV after the hemodialysis session [13]. We could not find increased levels of platelet-derived MV after hemofilter passage. This may be explained by the use of regional citrate anticoagulation in all patients, which has previously been demonstrated to result in significantly less platelet activation than heparin anticoagulation [6, 7].

### Correlation of filtration fraction with MV generation

Interestingly, we found correlations between FF and increments of platelet- and presumably leukocyte-derived as well as procoagulant AnnexinV+ MV. The usually recommended FF is below 25% in postdilution mode [22], since higher FF frequently leads to hemofilter clotting [5]. As shown in Fig. 2, the induction of MV generation within the hemofilter seems to be more pronounced when FF is above 30%, probably due to increased shear stress at higher FF. This prominent effect at higher FF seems even to apply to platelet-derived and AnnexinV+ MV which did not significantly change in septic patients overall. We speculate that the generation of MV within the hemofilter might contribute to hemofilter clotting occurring when high FF is applied. However, this remains to be investigated in a larger study.

Mechanisms contributing to the presumed cellular activation and the subsequent MV generation within the hemofilter might include a high shear stress or contact with the hemofilter membrane [5–7, 23–25]. Such effects of CVVH might even be more pronounced in a septic state where cells are already preactivated or highly susceptible to activation. Accordingly, septic shock patients exhibit already threefold higher levels of presumably mostly leukocyte-derived CD31+/CD41− MV compared to healthy subjects, as recently reported [19]. As calcium is a key mediator for neutrophil degranulation that is induced by dialysis membranes [23] and as influx of extracellular calcium is required for vesiculation of platelets induced by shear stress [24] one might speculate that MV release might even be more pronounced in CVVH without citrate anticoagulation [6], but this needs to be tested.

### Clearance of tissue factor by hemofiltration

Subsets of leukocytes, such as monocytes, are able to release MV and also associated TF upon activation [26, 27]. Based on the assumption that the above-mentioned increase in CD31+/CD41− predominantly reflects leukocyte activation we wanted to investigate whether we can find evidence for an accompanying TF generation during CVVH. Former studies reporting TF levels during hemofiltration analyzed exclusively systemic TF levels [28, 29]. Although Cardigan et al. [28] observed an increase in 50% of patients while on CVVH, no significant systemic increase could be detected in the most recent study from Bouman et al. [29]. So far, it was unclear whether potential alterations in systemic TF levels reflect secondary systemic inflammatory effects that are triggered by hemofiltration or if these alterations are due to TF generation or clearance within the hemofilter. Our study adds two new key answers to this matter: First, we provide evidence that hemofilter passage leads to a reduction in TF levels. Additionally, we demonstrate that a fraction of TF is being ultrafiltrated.

### Strengths and limitations

A major strength of our study is that we assessed alterations in MV levels occurring directly and exclusively within the hemofilter. Limitations of the study are the relatively small number of patients as well as the lack of information concerning kinetics in the extracorporeal system or the systemic circulation because we did not take serial measurements due to the complexity of analysis. Since no conclusion can be drawn regarding whether the generation of CD31+/CD41− MV within the hemofilter may impact systemic levels or patient outcome, the clinical implications of MV generation within the hemofilter remain to be further elucidated.

## Conclusions

In conclusion, CVVH clears a fraction of small PS-exposing MV by mechanisms other than filtration, but does not eliminate larger MV. CVVH induces the release of CD31+/CD41− MV that indicate leukocyte activation during hemofilter passage in septic patients. The generation of MV within the hemofilter seems to be particularly pronounced when FF is above 30%, which supports the clinical relevance of the usual recommendation to keep FF below 25%. A fraction of TF is being cleared by CVVH via ultrafiltration. Our findings show that MV analysis can be used to assess the effects caused by contact of blood with the hemofilter. Evaluation of MV patterns might be a promising instrument helping to refine extracorporeal therapeutic systems and biocompatibility of hemofilter membranes in future.

## Additional file

**Additional file 1: Fig. S1.** Workflow showing excluded parameters (filter survival and experimental data) relevant to final analyses. CVVH = Continuous veno-venous hemofiltration; MV activity = Microvesicle activity.

## Abbreviations

CVVH: continuous veno-venous hemofiltration (CVVH)
RRT: renal replacement therapy
MV: microvesicles
ICU: intensive care unit
SIRS: systemic inflammatory response syndrome
APCAHE II: acute physiology and chronic health evaluation score
SAPS II: simplified acute physiology score
SOFA: sequential organ failure assessment score
PFP: platelet-free plasma
PECAM: platelet endothelial cell adhesion molecule
GPIbα: glycoprotein Ib alpha
GPIIb: glycoprotein IIb
PBS: phosphate-buffered saline
CRP: C-reactive protein
PCT: procalcitonin
CEC: circulating endothelial cells
TAT: thrombin–antithrombin
FVII: factor VII
*C*_meas_: concentration of MV measured at specific port (MV/µl)
Amount_prefilter_: amount of MV entering the hemofilter (MV/min)
Amount_postfilter_: amount of MV leaving the hemofilter via blood (MV/min)
Amount_UF_: amount of MV leaving the hemofilter via ultrafiltrate (MV/min)
BF: blood flow (ml/min)
CF: flow of citrate-containing solution (ml/h)
UF: ultrafiltrate flow (ml/h)
FF: filtration fraction
