# Supplementary figures and images for: Hemofiltration induces generation of leukocyte-derived CD31+/CD41− microvesicles in sepsis
Source: Ann Intensive Care. 2017 Sep 4;7:89. doi: 10.1186/s13613-017-0312-3 (PMC5583134; doi:10.1186/s13613-017-0312-3)

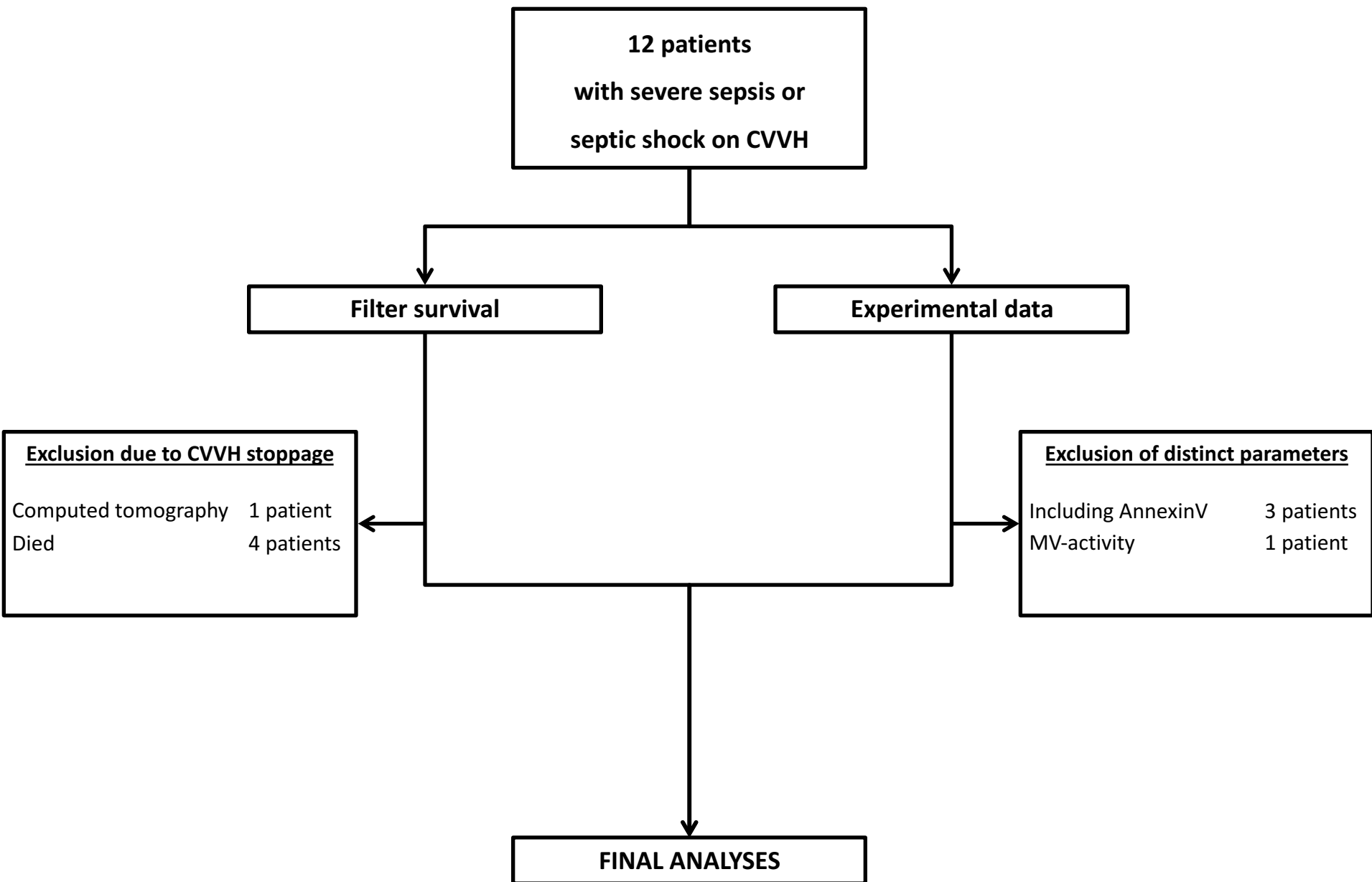

Supplement: Supplementary file 1 — Additional file 1: Fig. S1. Workflow showing excluded parameters (filter survival and experimental data) relevant to final analyses. CVVH = Continuous veno-venous hemofiltration; MV activity = Microvesicle activity. [file 13613_2017_312_MOESM1_ESM.pdf]
